# Efficacy of sarolaner (Simparic™) against induced infestations of *Amblyomma cajennense* on dogs

**DOI:** 10.1186/s13071-017-2324-0

**Published:** 2017-08-17

**Authors:** Fabio Scott, Lilian Franz, Diefrey Ribeiro Campos, Thaís Ribeiro Correia Azevedo, Daise Cunha, Robert H. Six, Steven Maeder, Travis Cree

**Affiliations:** 1 Programa de Pós-graduação em Ciências Veterinárias do Instituto de Veterinária da Universidade Federal Rural do Rio de Janeiro, DPA-IV-UFRRJ Br 467, Km 7, Seropédica, Rio de Janeiro, Brazil; 2 Zoetis, Veterinary Medicine Research and Development, Rua Luiz Fernando Rodriguez, Campinas, SP 1701 Brazil; 3 Zoetis, Veterinary Medicine Research and Development, 333 Portage St, Kalamazoo, MI 49007 USA

**Keywords:** *Amblyomma cajennense*, Brazilian spotted fever, Dog, Efficacy, Isoxazoline, Oral, *Rickettsia rickettsii*, Sarolaner, Simparic™, Simparica®, Tick

## Abstract

**Background:**

*Amblyomma cajennense* is the main vector of *Rickettsia rickettsii* which causes Brazilian spotted fever. This adult tick preferably infests horses and capybaras, but has low host specificity during its immature stages, thus posing a threat to humans and dogs. In this study, the efficacy of sarolaner (Simparic™**/**Simparica**®**, Zoetis) when administered once orally to dogs at 2 mg/kg was evaluated against induced infestations of *A. cajennense* nymphs for up to 35 days after treatment.

**Methods:**

Based on pretreatment tick counts, 20 dogs were randomly allocated to treatment with sarolaner (Simparic™) dosed at 2 mg/kg of body weight or a placebo on Day 0 of the study. Artificial infestations were performed using laboratory raised *A. cajennense* nymphs on study days -2, 5, 12, 19, 26 and 33. Efficacy was determined at 48 h post-treatment or post-infestation at each time point relative to the counts for dogs that received placebo.

**Results:**

There were no adverse reactions to treatment. A single dose of sarolaner (Simparic™) provided 100% efficacy on study days 2, 7 and 14; and ≥ 99.6% on days 21, 28 and 35. Geometric mean live tick counts for sarolaner were significantly lower than those for placebo on all days (*P* < 0.0001).

**Conclusions:**

Under the conditions of the present study, sarolaner (Simparic™) administered once orally at 2 mg/kg provided 100% efficacy against existing infestations and ≥ 99.6% efficacy within 48 h against weekly challenges of *A. cajennense* for at least 35 days after treatment.

## Background

*Amblyomma cajennense* or the Cayenne tick is a three-host ixodid tick species of low host specificity during its immature stages. This species is the main vector of *Rickettsia rickettsii* which causes Brazilian spotted fever, also known as Rocky Mountain spotted fever (RMSF) [1]*.* Other tick species, such as *Rhipicephalus sanguineus* (*sensu* *lato*) [2] and *Amblyomma aureolatum* [3], have been identified as potentially involved in the transmission cycle, although to a lesser extent. The agent of RMSF, *R. rickettsii*, is highly virulent to both humans and dogs [4]. Several cases of human infections have been preceded by RMSF in dogs in the United States [4–6], while four human deaths were reported in the State of Espírito Santo, Brazil in 1991 [7].

Serology of healthy dogs in Brazil has indicated past infection by *R. rickettsii* [3, 8] and has helped identify several endemic areas in the country. The estimated prevalence of antibodies against *R. rickettsii* in dogs ranged from 4.1 to 64% [9] and was demonstrated to increase with age [10]. Low specificity of the serological test hinders a more accurate epidemiological estimate. Some of the endemic areas in Brazil, from which the *R. rickettsii* has been isolated from the *A. cajennense* tick, include the states of Minas Gerais [1], São Paulo [11], Bahia, Goias, Rio Grande do Sul [12] and Espírito Santo [7]. Dogs infected with *R. rickettsii* may show non-specific clinical signs including fever, depression, anorexia, ocular lesions, hemorrhagic petechiae, anemia and thrombocytopenia [13]. All of these signs are also present in canine monocytic ehrlichiosis (CME) caused by another agent (*Ehrlichia canis*). Therefore, many clinical cases of Brazilian spotted fever are potentially being misdiagnosed as CME [4].

The adult stage of *A. cajennense* preferably feeds on horses and capybaras (*Hydrochoerus hydrochaeris*) [14]. With rapid urbanization these wild animals are seen in several non-rural settings, adapting easily and impacting the biology and ecology of the arthropod vectors, increasing the risk of exposure of dogs to vector-borne pathogens [15].

The life-cycle of *A. cajennense* lasts a year with population peaks in distinct seasons in Brazil with larvae most commonly found from March to July. The nymphal stage is found frequently during the months of July to October while adult forms are more common during the warmer months between September and March [16]. Larvae of *A. cajennense* can fast for 6 months in the environment and when attached to a host they feed for about 5 days, detach to search for shelters on the ground to then become nymphs. This can take 25 days, but might last as long as 1 year. After finding its second host, this stage feeds for 5 to 7 days and then detaches to undergo its second molt. After another 25 days, a young adult emerges ready to feed and breed on the third host. The adult stays on the host for about 10 days, and when the engorged female detaches to lay 5000 to 8000 eggs and start a new generation [17]. The transmission of *R. rickettsii* can be transovarian and transstadial. This allows the tick to remain infected with the bacterium for its whole life and to transfer it to the next generation [1].

The effective control of *A. cajennense* in dogs is vital due to several aspects of its biological cycle and its role in life-threatening diseases such as Brazilian spotted fever. Most tick control products for dogs are available as topical formulations, either as collars, shampoos, immersion baths or spot-on applications. Recently, some oral alternatives have also become available which can add convenience for the dog owner, leading to a higher compliance with administration.

Sarolaner is an acaricide and insecticide belonging to the novel isoxazoline group, which is available in a chewable tablet formulation (Simparic™, São Paulo, Brazil). It inhibits the function of the neurotransmitter gamma aminobutyric acid (GABA) receptor and glutamate receptor, acting in the neuromuscular junction in ticks and fleas, and thus, providing excellent control of fleas and ticks for at least 1 month after a single oral dose [18]. Simparic™ has proven to be effective against a wide range of ticks around the world [18–23]. No studies have previously been conducted to evaluate the efficacy of sarolaner against infestations of *A. cajannense* on dogs. The objective of this study was to evaluate the efficacy of sarolaner against existing *A. cajannense* infestations and weekly re-infestations for a period of 5 weeks following treatment with a single dose.

## Methods

The study was a masked, negative controlled, randomized, laboratory comparative efficacy study conducted at the Laboratory of Experimental Chemotherapy in Veterinary Parasitology (LQEPV) of the Federal Rural University of Rio de Janeiro, Brazil. Study procedures were in accordance with the World Association for the Advancement of Veterinary Parasitology (WAAVP) guidelines for evaluating the efficacy of parasiticides for the treatment, prevention and control of flea and tick infestation on dogs and cats [24], and complied with the principles of Good Clinical Practices [25]. Masking of the study was assured through the separation of functions. All personnel conducting observations or animal care or performing infestations and counts were masked to treatment allocation.

### Animals

Twenty beagle dogs (10 males and 10 females) from 10 months to 7 years of age and weighing from 8 to 15 kg were included in the study. Each dog was identified with an electronic transponder and had undergone a minimum of 60 days washout period to ensure that no residual ectoparasiticide efficacy remained from any previous treatment. Dogs were housed in individual outdoor kennels with cement walls and flooring that conformed to accepted animal welfare guidelines and ensured no direct contact between dogs. Dogs were acclimatized to these conditions for at least 14 days prior to treatment. Dogs were fed an appropriate maintenance ration of a commercial dry canine feed for the duration of the study. Water was available ad libitum. All dogs were given a physical exam to ensure that they were in good health at enrollment and suitable for inclusion in the study. General health observations were performed at least once daily throughout the study.

### Design

The study followed a randomized complete block design. Twenty dogs were ranked according to pre-treatment tick counts on Day -5 into blocks of 2, and within each block dogs were randomly allocated to treatment with either placebo or sarolaner, resulting in 10 dogs in each treatment group. Dogs were infested with ticks 2 days prior to treatment and then weekly on days 5, 12, 19, 26 and 33. Tick counts were conducted 48 h after treatment or re-infestation by counting live ticks present on Days 2, 7, 14, 21, 28 and 35.

### Treatment

Body weights taken on Day -2 were used to determine the appropriate dose to be administered. On Day 0, dogs allocated to T01 received placebo, while dogs of treatment group T02 received sarolaner. Each dose was calculated to provide the recommended dose of 2 mg/kg (range 2 to 4 mg/kg). Placebo and sarolaner tablet presentations were similar to maintain blinding.

All doses were administered by hand pilling to ensure accurate and complete dosing. Each dog was observed for several minutes after dosing for evidence that the dose was swallowed, and for general health at 1, 3, 6 and 24 h after treatment administration.

### Tick infestation and assessment

Adult engorged females of *A. cajennense* were collected directly from pastured horses in Rio de Janeiro and were raised in the laboratory to reach adequate numbers of nymphs for artificial infestation throughout the study.

Infestations were performed using the nymphal stage, because of its low host-specificity. Each dog was infested with approximately 200 *A. cajennense* nymphs. Dogs were restrained for about 5 min while the contents of the vial containing the live non-fed nymphs were deposited on their dorsum. For host suitability and allocation of the dogs into the study group this procedure was performed on Day -7 and tick counts conducted on Day -5. To assess efficacy against existing infestations, dogs were challenged on Day -2 (2 days before treatment). Subsequent weekly infestations occurred on study Days 5, 12, 19, 26 and 33. Tick counts were conducted 48 h after treatment and 48 h after each infestation (i.e. on study days 7, 14, 21, 28 and 35).

For tick removal and counts, dogs were systematically inspected so that the whole body surface was examined by hand and with the aid of a fine toothed comb. Each tick was manually removed and counted. If no ticks were found, systematic searching continued for an additional 5 min.

### Statistical analysis

The individual dog was the experimental unit and the primary endpoint was the live tick count. Data for post-treatment live (free plus attached) tick counts were summarized with arithmetic (AM) and geometric (GM) means by treatment group and time point. Tick counts were transformed by the log_e_(count + 1) transformation prior to analysis in order to stabilize the variance and normalize the data. Using the PROC MIXED procedure (SAS 9.3, Cary NC), transformed counts were analyzed using a mixed linear model. The fixed effects were treatment, time point and the interaction between time point and treatment by time point. The random effects included block, block by treatment interaction and error. Testing was two-sided at the significance level α = 0.05.

The assessment of efficacy for live ticks was based on the percent reduction in the arithmetic and geometric mean live tick counts relative to placebo, as suggested by the most recent guidelines of the WAAVP for systemic acaricides [24], and was calculated using Abbott’s formula:

$$ \%\mathrm{reduction}=100\times \left[\mathrm{mean}\  \mathrm{count}\ \left(\mathrm{placebo}\right)-\mathrm{mean}\  \mathrm{count}\ \left(\mathrm{treated}\right)\right]/\mathrm{mean}\  \mathrm{count}\ \left(\mathrm{placebo}\right) $$

## Results and discussion

There were no treatment-related adverse events during the study. Placebo-treated dogs maintained adequate tick infestations throughout the study with tick counts ranging from 24 to 83 (Table 1).

**Table 1:** Geometric (arithmetic) mean live *A. cajennense* counts and ranges for placebo and treated dogs and percent efficacy relative to placebo for dogs treated once orally with sarolaner chewable tables at 2 mg/kg on day 0 for evaluations performed at 48 h ﻿after treatment and post-treatment re-infestations

		Placebo	Sarolaner	% Efficacy				
	Day	Mean	Range	Dogs with ticks	Mean	Range	Dogs with ticks	
48 h post-treatment	2	47.0 (49.2)	27–73	0/10	0.0^a^ (0.0)	0–0	0/10	100
48 h post-infestation	7	50.5 (51.8)	32–68	0/10	0.0^a^ (0.0)	0–0	0/10	100
	14	48.2 (50.6)	28–73	0/10	0.0^a^ (0.0)	0–0	0/10	100
	21	54.0 (56.2)	34–83	0/10	0.1^a^ (0.1)	0–1	1/10	9.99
	28	53.2 (56.4)	27–80	0/10	0.1^a^ (0.2)	0–1	2/10	7.99
	35	51.5 (54.8)	24–78	0/10	0.2^a^ (0.3)	0–1	3/10	6.99

^a^Geometric mean live tick count significantly lower than placebo (*P* < 0.0001)

For the sarolaner treated group, no live ticks were found on any dog on Days 2, 7 and 14. On Day 21, one tick was found on one dog, on Day 28, two of the 10 dogs had one tick each, and on Day 35 three of the ten dogs had one tick each. Therefore, the percent reduction in arithmetic mean live tick count compared to placebo was 100% on Days 2, 7 and 14; 99.9% on Day 21; 99.7% on Day 28; and 99.6% on Day 35. Geometric mean counts for dogs in the sarolaner group were significantly lower (t_(14.9)_ = 38.71, *P <* 0.0001) than the placebo group at all the time points (Table 1).

To our knowledge this the first reported study evaluating efficacy of an oral acaricidal product against *A. cajennense* on dogs. The efficacy observed against *A. cajennense* in this study, 100% within 48 h of treatment of an existing infestation, and ≥ 99.6% within 48 h of weekly re-infestation for 35 days, is comparable with the efficacies reported for sarolaner against other tick species commonly found on dogs. Six et al. [21] showed that sarolaner provided ≥ 99.6% efficacy within 48 h of treatment and ≥ 99.6% efficacy within 48 h after weekly re-infestation for 35 days against *Amblyomma americanum*, *Amblyomma maculatum*, *Dermacentor variabilis*, *Ixodes scapularis* and *Rhipicephalus sanguineus*. Similarly, Geurden et al. [23] showed that sarolaner provided ≥ 99.7% efficacy within 48 h of treatment and ≥ 97.5% efficacy within 48 h after weekly re-infestation for 35 days against *Dermacentor reticulaus*, *Ixodes hexagonus*, *I. ricinus* and *R. sanguineus.*

The present study evaluated efficacy against nymphal ticks, as that is the stage of *A. cajennense* most commonly infesting dogs. The studies reported by Six et al. [18–22] and Geurden et al. [22, 23] evaluated efficacy against adult ticks. It is interesting to note that the efficacy of sarolaner demonstrated against adult ticks was similar to that observed against nymphal *A. cajennense*, given that the immature stages are generally considered to be more susceptible than adults [26].

An initial attachment and feeding period of at least 24 to 48 h is required before transmission of many tick-borne pathogens can occur [27, 28], and if ticks are killed within that time, transmission may be prevented [29]. Pathogen transmission times reported from studies that have specifically evaluated *R. rickettsii* transmission from tick vectors to mammalian hosts vary considerably [30]. The variation in transmission times reported in these studies is most likely due to the variability in specific study conditions, such as the number of infected ticks applied, rate of infection in applied ticks, and the previous feeding status of ticks. However, Hayes et al. [31] showed that *D. andersonii* infected with *R. rickettsii* required warming at elevated temperatures (37 °C) for 24 to 48 h, or blood-feeding of greater than 10 h for the *R. rickettsii* to become virulent. Although specific pathogen transmission model studies would be needed for confirmation, existing data seem to support that killing *A. cajennense* within 48 h should reduce the risk of *R. rickettsii* transmission to dogs.

## Conclusions

This study confirms the acaricidal efficacy of sarolaner (Simparic™) against an existing *A. cajennense* infestation after a single oral dose of 2 mg/kg and the sustained control for up to 35 days post-treatment. The convenient chewable oral formulation offers a valuable tool for the treatment of tick infestations and potentially for the prevention of tick-borne diseases in dogs.

## Abbreviations

AM: Arithmetic mean
CME: Canine monocytic ehrlichiosis
GABA: Gamma aminobutyric acid
GM: Geometric mean
RMSF: Rocky Mountain spotted fever
